# Assisting manual literature curation for protein–protein interactions using BioQRator

**DOI:** 10.1093/database/bau067

**Published:** 2014-07-21

**Authors:** Dongseop Kwon, Sun Kim, Soo-Yong Shin, Andrew Chatr-aryamontri, W. John Wilbur

**Affiliations:** ^1^Department of Computer Engineering, Myongji University, Yongin 449-728, South Korea, ^2^National Center for Biotechnology Information, National Library of Medicine, National Institutes of Health, Bethesda, MD 20894, USA, ^3^Department of Biomedical Informatics, Asan Medical Center, Seoul 138-736, South Korea and ^4^Institute for Research in Immunology and Cancer, Université de Montréal, Montréal QC H3C 3J7, Canada

## Abstract

The time-consuming nature of manual curation and the rapid growth of biomedical literature severely limit the number of articles that database curators can scrutinize and annotate. Hence, semi-automatic tools can be a valid support to increase annotation throughput. Although a handful of curation assistant tools are already available, to date, little has been done to formally evaluate their benefit to biocuration. Moreover, most curation tools are designed for specific problems. Thus, it is not easy to apply an annotation tool for multiple tasks. BioQRator is a publicly available web-based tool for annotating biomedical literature. It was designed to support general tasks, i.e. any task annotating entities and relationships. In the BioCreative IV edition, BioQRator was tailored for protein– protein interaction (PPI) annotation by migrating information from PIE *the search*. The results obtained from six curators showed that the precision on the top 10 documents doubled with PIE *the search* compared with PubMed search results. It was also observed that the annotation time for a full PPI annotation task decreased for a beginner-intermediate level annotator. This finding is encouraging because text-mining techniques were not directly involved in the full annotation task and BioQRator can be easily integrated with any text-mining resources.

**Database URL:** http://www.bioqrator.org/

## Background

Although high-throughput technologies such as microarrays and next-generation sequencing have produced a large amount of biological data, much valuable information is mostly available in biomedical literature, not in computer-parsable forms. Hence, developing biological databases by curating literature has been highlighted as a major research topic (1, 2). It is widely known that manual curation is the most accurate way to annotate literature; however, annotation (annotation and curation are different concepts in a strict sense. However, in this article, the terms are used interchangeably) is expensive in regard to both financial expense and time (3). In addition, the rapid growth of biomedical literature makes it difficult to manually extract all the available information (4). To overcome the pitfalls of manual annotation, some attempts have been made to generate annotations using text-mining techniques (5–7). Nevertheless, state-of-the-art text mining still cannot replace humans; thus text-mining-assisted curation is a promising and practical solution to address the manual curation issue (8–11).

A biocuration workflow consists of the following different stages in general (2, 12):

- Triage: finding relevant articles for curation.
- Bio-entity identification and normalization: identifying mentions of relevant bio-entities and linking the entities to database identifiers.
- Annotatable event detection: identifying relevant events for annotation such as protein–protein interactions (PPIs).
- Evidential qualifier association: finding experimental evidence supporting annotated events.

Furthermore, the tools that support manual annotation should be intuitive to use, should include visualization of annotated text and should support easy-to-parse input and output formats (13, 14). To summarize, an annotation tool should be able to support a few or all stages listed above, but in a user-friendly interface. These general requirements were our main interest in developing an annotation interface because most curation systems currently available solve very specific problems and few of them can be used for multiple annotation tasks (14–16).

BioQRator (17) is a general-purpose user interface for annotating bio-entities and relationships. Simple and minimal network messages are used to communicate between BioQRator and text-mining resources. This enables one to easily create a customized interface for any bio-curation project if the task involved is to annotate entities and/or relationships. An important issue for curation systems is the multiple different formats that are used. To address this problem, we adopt BioC (18, 19) as a standard input and output format. For input, BioC formatted documents or PubMed abstracts can be used. For output, annotated documents can be saved in the BioC or CSV (comma-separated values) format as well. More importantly, BioQRator provides an easy-to-use interactive web interface. It also supports multiple browsers including Chrome, Firefox and Safari (partially compatible with Internet Explorer).

The BioCreative Interactive task (IAT) is a track designed for exploring user–system interactions, promoting development of useful text-mining tools and providing a communication channel for biocuration and text-mining communities (20, 21). For BioCreative IV, a participating team defined a topic and tasks for curators’ evaluation. Biocurator volunteers were assigned to participating teams based on their interests in defined topics. The BioCreative IAT task is especially meaningful for text-mining communities because there has been little effort to formally evaluate curation tools. To demonstrate the usability of BioQRator in BioCreative IV, a PPI task was defined and PIE *the search* (22, 23) was used for an external text-mining resource. Necessary databases such as PubMed, Entrez Gene and UniProt were used to provide web links for BioQRator.

To evaluate the performance of BioQRator, two tasks were proposed. Task I was to evaluate the ability of PIE *the search* to rank relevant articles high in the output list processed by BioQRator. Task II was to measure annotation time and users’ experience on the BioQRator interface. A total of six curators from BioGRID (http://thebiogrid.org), MINT (http://mint.bio.uniroma2.it) and TRIP (http://trpchannel.org) databases volunteered. Five curators participated in Task I and four curators participated in Task II. The experimental results show that, for Task I, the customized BioQRator provided 63% precision, whereas the PubMed search provided 29% precision for the top-ranked 10 documents. For Task II, the average annotation time was not changed much overall between manual and BioQRator annotations; however, significant time was saved for the annotation task done by a beginner-intermediate level curator. However, the ratings of overall satisfaction from curators’ feedback were all either positive or very positive (21).

## Methods

### BioQRator

**Figure 1.**
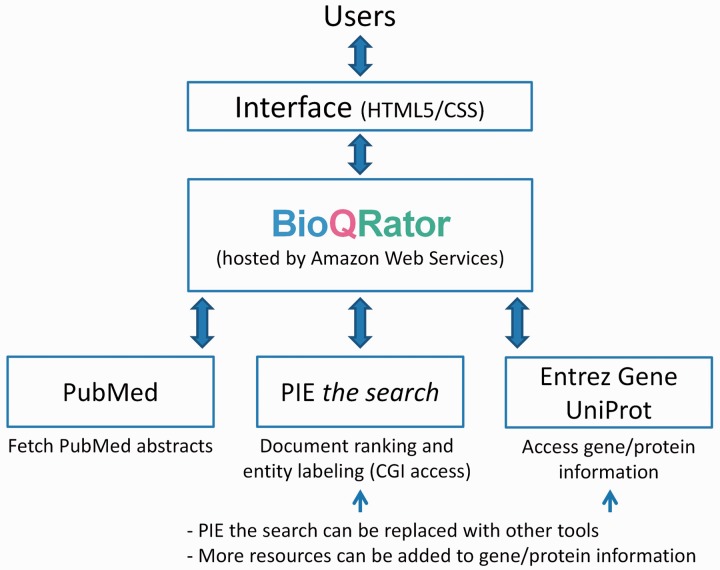
Overall framework of BioQRator in default setting. BioQRator supports an annotation interface to highlight entities and relations. The default setup uses PIE *the search* as a triage and an entity labeling module and Entrez Gene and UniProt are used for recommending and linking IDs. BioQRator was designed to use any BioC document; however, the current interface basically assumes all documents are PubMed abstracts.

Our initial focus for developing BioQRator was to create a general-purpose annotation tool for entities and relationships. BioQRator is essentially a web interface that can be fully customized for a given task. Using this interface, most annotations can be done by a series of single mouse clicks or drags with simple typing. Figure 1 shows the overall framework of the BioQRator system. The HTML5/CSS implementation allows BioQRator to support multiple browsers such as Chrome, Safari and Firefox (partially compatible with Internet Explorer). BioQRator relies on e-utils (http://www.ncbi.nlm.nih.gov/books/NBK25501) to fetch PubMed abstracts. As a PPI task was defined for BioCreative IV IAT, PIE *the search* was utilized for ranking PubMed abstracts and labeling protein names. For linking bio-entities to biological databases, i.e. entity normalization, Entrez Gene and UniProt were set up as default; however, additional databases can be added as well through the web interface. Following are the basic functionalities BioQRator provides.

- Handling documents based on a collection: a user creates a collection for an annotation project and adds documents to the collection.
- Adding documents by PubMed queries, PubMed IDs (PMIDs) or BioC files: users can add PubMed abstracts via an external triage module, e.g. PIE *the search*. BioC files are also allowed for input. This is particularly useful when pre-annotated documents are ready and available to use.
- Use of the smart document finder: this is a convenient tool for periodically adding documents with a fixed PubMed query. A user will be able to set automatic document search weekly, monthly, quarterly or yearly.
- Creating entities (To be compatible with the BioC format, the web interface of BioQRator displays an entity as an annotation. This is because any text can be annotated in BioQRator, and BioC marks text as an annotation) and relation types: as a general-purpose tool, a user can create any type of entities and relations in BioQRator. A user also can switch to different external resources through the ‘Recommenders’ tab in the web interface (Default resources: Entrez Gene and UniProt).
- Use of recommenders for Entrez Gene and UniProt IDs: For normalizing gene/protein names, Entrez Gene and UniProt searches are provided through recommenders. We made the normalization process easier by providing an additional pop-up menu to recommend Entrez Gene/UniProt IDs for a selected text.
- Share a collection: A collection can be shared with other users. This function is enabled if other users are added through the ‘Share’ button for a collection.
- Download a collection: Annotated documents in a collection can be saved as either a BioC or CSV file. BioC was developed to easily share text documents and annotations among different tools. Since BioCreative IV took the BioC initiative as one of its main tasks, we decided to fully support BioC as the standard input and output file format.

### Communication between BioQRator and text-mining modules

The tasks BioQRator covers in the biocuration workflow are ranking documents (Triage Module) and annotating entities/relationships (Entity/Relation Module). As BioQRator requires external text mining resources to obtain this information, three message formats were defined for communicating between BioQRator and text-mining tools.

**Figure 2.**
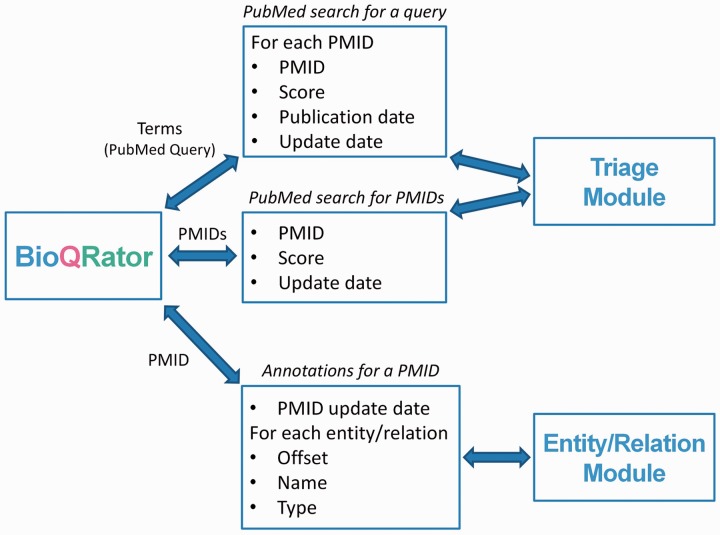
Message formats for communicating with BioQRator. To integrate BioQRator with text mining resources, two external modules, i.e. triage and entity/relation modules, are necessary. The triage module receives PubMed queries or PMIDs as a request and returns PMIDs and their corresponding scores. For entities and relations, BioQRator sends a PMID and the external module returns offsets, entity/relation names and types.

Figure 2 explains what messages are used and what information is sent though a remote network connection. For triage of PubMed abstracts, BioQRator supports searches by PubMed queries and PMIDs. For ‘search by PubMed query’, a query is sent to a Triage Module and PMIDs, scores (e.g. PPI scores by PIE *the search*), publication dates and update dates (the latest revision date for PubMed documents) are returned. For ‘search by PMIDs’, the message format is simpler. Only PMIDs, scores and update dates are returned as a result. If entity and/or relation prediction modules are available, they can be used to highlight entities and/or relations in BioQRator. For a PMID, the Entity/Relation Module returns the PMID update date, offsets, names and types.

A Triage Module is required for PubMed search because it is necessary to show a ranked list of PMIDs. However, an Entity/Relation Module is optional and only necessary if a user wants to use a prediction tool.

### PIE *the search*

Our second goal in BioCreative IAT was to evaluate the practical usability of PIE *the search* in the bio-curation workflow. PIE *the search* is an article ranking engine for searching PubMed literature for PPI information, and the main approach is based on a top-performance method in BioCreative III (23). For BioCreative IV IAT, we integrated BioQRator with PIE *the search* for obtaining document ranking information and some protein names.

**Figure 3.**
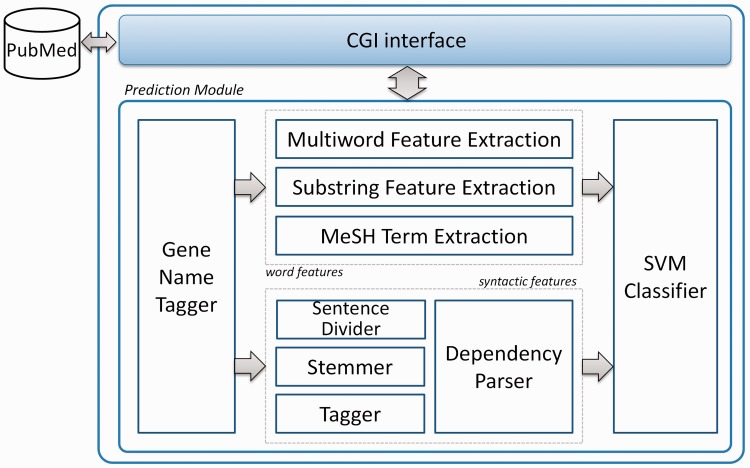
Overview of PIE *the search*. In PIE *the search*, input articles are evaluated whether there are gene/protein names in the text. After gene name detection, word and syntactic features are created. An SVM classifier is used for classifying PubMed abstracts. This diagram was modified from [22].

PIE *the search* is a machine-learning framework to classify PPI informative documents. Input articles are evaluated whether there are gene/protein names in the text. After gene-name detection, features are created in two different ways: word features including multiwords, substrings and MeSH terms; syntactic features involving grammar relations between words. Finally, a support vector machine (SVM) classifier with the modified Huber loss function (24) is used for classifying documents. In previous work (23), we evaluated article ranking performance using the BioCreative III ACT (BC3) data set (4). For F1, MCC (MCC: Matthews’ correlation coefficient) and AUC iP/R (AUC iP/R: the area under the interpolated precision and recall curve) measures, PIE *the search* showed 0.6258, 0.5610 and 0.6834, respectively. However, the medians of BC3 participant results were 0.5353 F1, 0.4563 MCC and 0.5367 AUC iP/R. For identifying gene/protein names, the Priority Model (25) is utilized in PIE *the search*. Because not all entities are important in PPI annotations, we only use predicted gene/protein names that are used to identify PPI informative articles. Figure 3 depicts the overall structure of PIE *the search*.

## Results and discussion

For BioCreative IV IAT, two subtasks were defined. Task I is to search and compare results from BioQRator and PubMed for evaluating triage for PPIs. Task II is to curate PPI-relevant interactions and normalize protein names for PubMed abstracts.

### Task I: document ranking performance

Five curators participated in Task I and each curator evaluated 10 top-ranked documents for 10 queries. Queries were chosen by curators based on their interests. However, they were asked to choose five queries from protein names and five queries from general topics, e.g. cystic fibrosis. Although any PPI informative article was treated as relevant for general topic queries, for protein queries, only the articles including the proteins in the query as PPIs were considered as relevant. Furthermore, the organizer argued that it was not fair to use the same query for both BioQRator and PubMed because PubMed was not developed for PPI article search. Thus, curators were allowed to add additional keywords such as ‘interact*’ and ‘bind*’ for PubMed searches.

**Figure 4.**
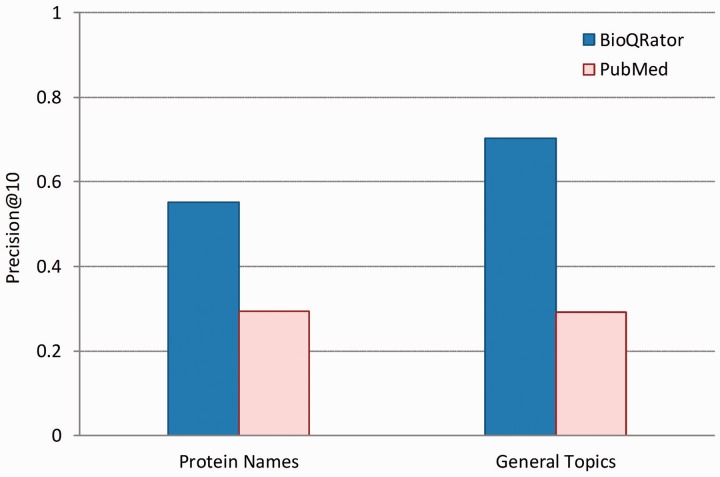
Performance comparison for top-ranked 10 documents from BioQRator and PubMed. The graph shows precision@10 for BioQRator and PubMed in protein name and general topic queries. Precision@10 is the precision at rank 10 on average.

Figure 4 shows the performance comparison for 10 top-ranked documents obtained from BioQRator and PubMed. Precision@10 is the precision at rank 10 on average. In this figure, BioQRator achieved 55.20% and 70.31% precision@10 for protein name and general topic queries, respectively. Meanwhile, PubMed achieved 29.33% and 29.20% precision@10 for the same query set. This evidence is more compelling considering additional interacting terms were used for PubMed search. Note that the performance difference is greater in general topics. This is due to the strict guideline applied for evaluating protein queries.

### Task II: PPI curation time and user experience

Task II was to perform PPI curation either manually or using BioQRator. The thorough assessment of annotation tools remains an arduous task for various reasons. First, consolidated metrics taking into account all the different parameters that influence curation efficiency are not yet available. Second, it is difficult to recruit experts for the time required to fully learn and exploit the curation platforms. Nevertheless, it appears clear that the ultimate goal of such tools is to increase the curation capacity of individual annotators.

Database curators usually rely on customized curation interfaces, and the level of familiarity with these clearly impacts curation speed. Therefore, to minimize the bias in the evaluation arising from interface familiarity, we opted to compare literature curation performed with BioQRator to annotation relying on a simple spreadsheet. The task required the annotation of PPIs from a list of 50 abstracts (25 PMIDs for manual annotation and 25 PMIDs for BioQRator annotation). Protein interactors had to be normalized to the appropriate gene or protein identifier (the choice was left to curators). Four curators participated in Task II, and 50 PubMed abstracts were randomly chosen from the PPI-relevant PMIDs annotated in Task I. The biocurators who produced the selected PMID set did not participate in Task II. Because of the difficulty of choosing an appropriate performance measure for annotation interfaces (as described above), the only parameter taken into account for Task II was the time employed to complete the curation of 50 abstracts. Although we did not expect annotation time would be significantly reduced without an intensive use of text mining tools, we hoped an easy-to-use interface in BioQRator would help curation speed.

**Figure 5.**
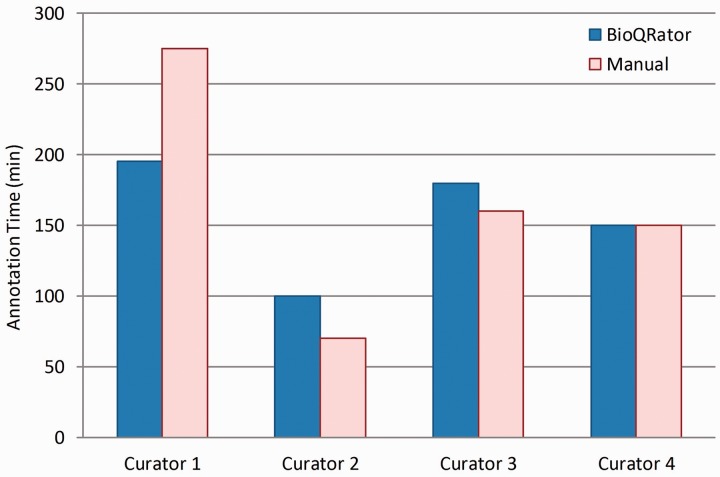
Comparison of times spent for BioQRator and manual curation. Four curators performed a full annotation task for 50 PubMed abstracts. Half were annotated using BioQRator and the other half using a spreadsheet. The split was random and changed for each curator. The bar graph here shows the total minutes each curator spent for annotation.

Figure 5 shows the comparison of times spent for BioQRator and manual curation. As expected, we did not observe any significant difference in the productivity of annotators when comparing the time necessary to annotate the provided literature in BioQRator vs. in spreadsheet files. However, a significant time was saved for Curator 1. From user feedback, Curator 1 labeled himself as a beginner-intermediate level curator. As a result, we may conclude that the current BioQRator interface guides annotation better for curators who are still in a learning phase.

We must note that curators usually tend to complete the evaluative task in the shortest possible time because these assignments are additional work to be done on top of a full day’s work. Hence, we suspect the intensity of effort in completing the task was higher than the one normally employed in the daily curation routine. It is also possible that annotation of information from abstracts is not the most appropriate task because the bulk of the curation effort is in scrutinizing the full paper.

Through Tasks I and II, we were not really able to discern the advantage provided by BioQRator in terms of managing the literature and easily tracking annotation progress. However, the user survey shows that all curators participating in this task were positive or very positive in overall satisfaction (21). The criteria of overall satisfaction include the rating of the user experience, the rating of the system and the recommendation of the system. One curator also commented that he found the environment very easy and relaxing, diminishing the stress of curation.

## Conclusions

We here present a web-based user interface, BioQRator, and its application to PPI annotation for BioCreative IV IAT. BioQRator was designed to annotate entities and relationships from literature available in PubMed or from any document formatted in the BioC standard. BioQRator is flexible so that any text-mining resource can be integrated via network communication. For the user study in BioCreative IV, a PPI task was defined and PIE *the search*—a text-mining engine for article ranking—was used as an external resource. For a PPI triage task, BioQRator with PIE *the search* produced 63% precision, whereas PubMed produced 29% precision at top-ranking documents. The full annotation task for PPI showed no significant differences between manual and BioQRator annotation times. However, the time was reduced by 30% for a beginner-intermediate level curator. As no text mining was directly involved in annotating proteins and PPIs, this demonstrates that BioQRator provides an intuitive and easy-to-use environment for bio-curation. The survey performed after evaluation also indicates that biocurators have positive or very positive satisfaction overall.

Inspired by the promising evidence obtained from BioCreative IV IAT, we plan to revise BioQRator to increase the flexibility as a generally applicable annotation tool. Tailored modules for specific annotation tasks, which currently can be established only by system developers, will be implemented in a web setting that can switch text-mining resources in a more convenient way. It also seems curation teams for biological databases prefer having an annotation interface locally. This can be done by creating a stand-alone package, which also remains as future work.
